# De-escalating axillary surgery in early breast cancer: external validation of a retrospective cohort

**DOI:** 10.1007/s00432-026-06498-6

**Published:** 2026-06-04

**Authors:** Mathilde Landry, Amelia Favier, Feriel Ksantini, Alexandra Gabro, Geoffroy Canlorbe, Catherine Uzan

**Affiliations:** 1 Department of Gynecological and Breast Surgery and Oncology, Assistance Publique des Hôpitaux de Paris (AP-HP), Pitié- Salpêtrière, University Hospital, 75013 Paris, France; 2 Sorbonne Université, INSERM, Unité Mixte de Recherche Scientifique 938 et SIRIC CURAMUS, Centre de Recherche Saint-Antoine (CRSA), Equipe Instabilité des Microsatellites, Paris, France; 3 AP-HP, University Institute of Cancer (IUC), Sorbonne University, Paris, France; 4 Department of Medical Oncology, Assistance Publique-Hôpitaux de Paris (AP-HP), Institut Universitaire de Cancérologie, Sorbonne Université, Pitié- Salpêtrière Hospital, Paris, France; 5 Radiation Oncology Department, La Pitié Salpêtrière University Hospital, Assistance Publique des Hôpitaux de Paris.Sorbonne University, Paris, France; 6 Saint-Antoine Research Center, Cancer Biology and Therapeutics, INSERM UMR_S_938, Sorbonne University, Paris, France; 7 Resident in Obstetrics and Gynecology, Pitié-Salpetriere University Hospital, Paris, France

**Keywords:** Early breast cancer, Sentinel lymph node biopsy, Axillary staging, Adjuvant chemotherapy

## Abstract

**Background:**

Sentinel lymph node biopsy (SLNB) is the standard for axillary staging in early breast cancer, yet its relevance is increasingly questioned in patients with favorable tumor biology when nodal status does not influence systemic treatment decisions.

**Methods:**

We conducted a retrospective, single-center observational study including 162 women with clinically and sonographically node-negative (cN0) pT1 invasive breast cancer treated between 2019 and 2024 at a French university hospital. All patients underwent axillary staging and had complete molecular profiling. Among the 25 patients with pathologically positive nodes (pN+), two multidisciplinary team (MDT) discussions were simulated: one blinded to nodal status (limited-information MDT) and one with complete clinicopathologic data (full-information MDT). Recommendations for adjuvant chemotherapy and genomic testing (Oncotype DX) were compared.

**Results:**

Most tumors were hormone receptor–positive (90%) and HER2-negative (87%); 9% were triple-negative. Despite negative preoperative axillary ultrasound, nodal involvement was identified in 25 patients (15%). In the limited-information MDT, chemotherapy was recommended in 8 patients and genomic testing in 11 patients. In contrast, the full-information MDT recommended chemotherapy in 16 patients and genomic testing in 9 patients, with 2 additional patients ultimately receiving chemotherapy. Omission of nodal status during MDT deliberation was associated with a significant reduction in chemotherapy recommendations (*p* < 0.01). Genomic testing contributed to treatment de-escalation in both scenarios.

**Conclusions:**

In selected patients with pT1 breast cancer, omission of axillary nodal status during MDT decision-making may safely reduce adjuvant chemotherapy use without compromising treatment planning, supporting current axillary de-escalation strategies.

## Introduction

Breast cancer is the most common cancer among women and represents a major public health issue, affecting approximately one in eight women over their lifetime (Dabakuyo-Yonli and Arveux 2020). T1 breast cancers are associated with an excellent prognosis, with breast cancer–specific survival exceeding 95% at 10 years; however, recurrence rates vary substantially according to molecular subtype, reflecting tumor biological heterogeneity (Zhu et al. 2021). In triple-negative tumors, recurrence rates may reach up to 25%, whereas they remain below 5% in luminal A cancers, highlighting the prognostic value of tumor biology beyond tumor size. Despite declining mortality, treatment-related morbidity remains a significant concern for patients’ quality of life.

Axillary lymph node involvement is a key determinant of treatment decisions in breast cancer. Patients with T1 disease are generally treated with breast-conserving surgery, sentinel lymph node biopsy (SLNB), radiotherapy, and systemic therapy adapted to tumor biology (Banys-Paluchowski et al. 2022). In hormone receptor–positive tumors, adjuvant chemotherapy decisions increasingly rely on genomic assays such as Oncotype DX (Puppe et al. 2020) although nodal status continues to influence genomic risk stratification. Concurrently, axillary lymph node dissection is increasingly avoided, even in selected patients with multiple positive nodes, in favor of less invasive approaches when systemic therapy or radiotherapy is planned (Prathibha et al. 2024), in order to reduce treatment-related morbidity without compromising oncologic outcomes (Giuliano et al. 2017).

In this context, accurate staging remains essential for patient stratification and the implementation of risk-adapted therapies (Gálvez et al. 2020). However, recent randomized trials have questioned the need for routine axillary staging in selected patients. The SOUND trial demonstrated that omission of axillary surgery in patients with tumors up to 2 cm and negative preoperative axillary ultrasonography was non-inferior to SLNB in terms of distant disease-free survival (Gentilini et al. 2023). Similarly, the INSEMA trial reported comparable invasive disease-free survival with fewer postoperative complications when surgical axillary staging was omitted (Reimer et al. 2025).

This study aimed, first, to evaluate whether therapeutic management, particularly the indication for chemotherapy, is influenced by sentinel lymph node analysis. And second, to validate our real-life cohort by performing an external validation of previously published findings within our institution.

## Methods

This was a single-center retrospective observational study conducted in the Department of Gynecological Surgery at La Pitié-Salpêtrière University Hospital (AP-HP) between January 2019 and January 2024. The study was conducted in accordance with the RECORD guidelines using data extracted from the AP-HP clinical data warehouse through the Cohort360 query system.

Patients were identified using predefined criteria including T1 breast cancer and surgical management within our department. All molecular subtypes were eligible for inclusion.

### Study population

Inclusion criteria were women aged ≥ 18 years who underwent surgery for pT1 invasive breast cancer with sentinel lymph node biopsy (SLNB) and had a negative preoperative axillary ultrasonography. In cases of suspicious lymph nodes, cytological biopsy was performed to exclude nodal involvement. All patients provided informed consent.

Exclusion criteria included distant metastases, biopsy-proven axillary involvement, neoadjuvant therapy, extensive multifocal or multicentric disease, bilateral breast cancer, prior ipsilateral breast cancer, and pregnancy.

### Treatment

A limited-information multidisciplinary team meeting (MDT) was conducted to evaluate the impact of sentinel lymph node status on adjuvant treatment decisions. The MDT consisted of three surgeons, one medical oncologist, and one radiation oncologist. All cases were anonymized, and nodal status was concealed to minimize recognition and anchoring bias.

The MDT was allowed to recommend one of three options: adjuvant chemotherapy, no chemotherapy, or use of a genomic assay.

In our institution, we use the Oncotype DX assay as part of our clinical decision-making. According to the TAILORx study, chemotherapy is indicated for premenopausal women with a recurrence score greater than 15, and for postmenopausal women with a score greater than 25.

We presented the 25 node-positive patients during a limited-information MDT. During this review, the specialists were blinded to the nodal status when making their therapeutic recommendations.

After the blinded review of each case, the conclusion of the limited-information MDT was either chemotherapy, an oncotype test, or no intervention.

To simulate therapeutic recommendations in the absence of SLNB, a retrospective blinded analysis was performed, assuming node-negative status. The UK PREDICT tool was used to estimate 10-year survival benefit from third-generation chemotherapy and to support decisions regarding genomic testing or chemotherapy in cases of clinical uncertainty. A predicted survival benefit ≥ 2% supported the indication for Oncotype DX testing, while a benefit ≥ 5% supported the recommendation for adjuvant chemotherapy.

### Outcome

The primary outcome was to assess the prevalence of adjuvant chemotherapy in patients with unknown axillary surgical staging. The secondary outcome was to evaluate the use of the Oncotype DX test in this setting.

### Complications

Postoperative complications were systematically recorded and classified as short-term (≤ 30 days) or long-term (> 30 days), and graded according to severity using a standardized classification (Dindo et al. 2004). Short-term complications included wound infection, hematoma, seroma, delayed healing, and early lymphedema. Long-term complications included chronic lymphedema, sensory neuropathy, chronic pain, breast fibrosis, and shoulder mobility impairment.

### Statistical analysis

We used the R Studio software to describe frequencies, percentages, medians, and IQRs. All tests were two-sided, and P-values less than 0.05 were considered statistically significant. Differences in the distribution of categorical variables were assessed using the χ2 test.

Indications of adjuvant chemotherapy and Oncotype DX test were compared between our blinded limited-information MDT recommendation and full-information MDT real decision, using Fisher’s exact test.

### Ethics

The study protocol was approved by the Ethics Committee of CEROG (Collège d’Évaluation des Recherches en Obstétrique et Gynécologie) (approval number: 2025-GYN-0708). All methods were performed in accordance with the relevant guidelines and regulations (Dabi et al. 2022).

## Results

### Patient characteristics

**Fig. 1:**
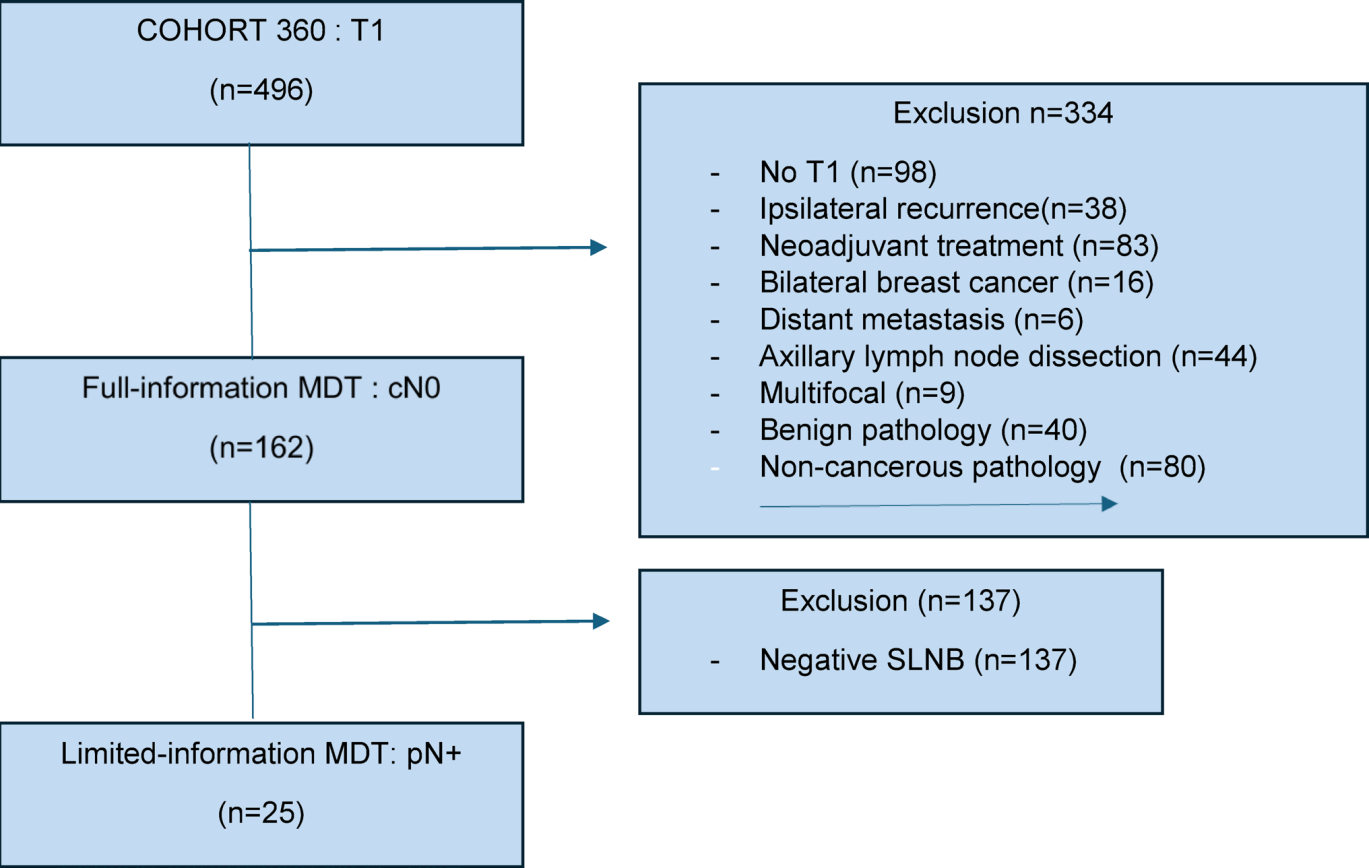
Flow chart.* Abbreviations*: SLNB= sentinel lymph node biopsy MDT= Multi disciplinary team T1 = breast tumor size 2 cm or less

The Cohort 360 platform initially identified 496 patients who met both predefined criteria (pT1 breast cancer and surgical management in our department), from which 162 were included in the final analysis after applying exclusion criteria. Patient’s characteristics were similar in both treatment arm. Flow chart is presented in Fig. 1.

 Baseline patients and tumor characteristics were similar between treatment groups.

 The median age at diagnosis was 59.0 years, with 67.9% of patients being peri- or postmenopausal. Most patients presented with low-risk breast carcinoma: 90.2% had estrogen receptor (ER) positive tumors, including 43.2% with ER positive and PR positive tumors (luminal A) and 33.9% with ER positive and PR negative tumors; 13% had ERBB2 overexpression and 19.8% had grade 3 tumors. The majority (75.9%) had an invasive carcinoma of no special type, and 66% had Ki-67 values < 20%. The majority of tumors were classified as pT1c, accounting for 61.7% of cases.

 More aggressive tumor subtypes were present with 9.2% triple-negative and 12.9% HER2-amplified.

In the population, 84.6% had a negative sentinel lymph node. Among those with a positive sentinel node, 13.6% had only one positive node, and 4.9% presented with micrometastases.

 Patient and tumor characteristics are presented in Table 1.

**Table 1:** Population’s characteristics

Characteristics	Patients, *n* = 162 (%)	N0 *n* = 137 (%)	N + *n* = 25(%)
*Age at surgery*	Median age: 59 Range, 28–89		
< 40	13 (8.02)	11 (8)	2 (8)
40–49	30 (18.5)	22 (16.1)	8 (32)
50–64	66 (40.7)	59 (43.1)	7 (28)
> 65	53 (32.7)	45 (32.8)	8 (32)
*Menopausal statuts*			
Premenopausal	52 (32.1)	41 (29.9)	11 (44)
Perimenopausal or postmenopausal	110 (67.9)	96 (70.1)	14 (56)
*Histotype*			
Ductual	123 (75.9)	103 (75.2)	20 (80)
Tubular	1 (0.6)	1 (0.7)	0
Lobular	25 (15.4)	25 (18.2)	0
Other	13 (8)	8 (5.9)	5 (20)
*Pathological tumor size*			
pT1a	15 (9.2)	14 (10.2)	1 (4)
pT1b	46 (28.3)	38 (27.7)	8 (32)
pT1c	100 (61.7)	84 (61.3)	16 (64)
*ER statut*			
0	16 (9.8)	14 (10.2)	2(8)
>=10	146 (90.2)	123 (89.8)	23 (92)
*PgR status*			
0	59 (36.4)	51 (37.2)	8 (32)
>=10	103 (63.6)	86 (62.8)	17 (68)
*ERBB2 overexpression*			
over	21 (13)	18 (13.1)	3 (11.5)
not over	141 (87)	118 (86.1)	22 (84.6)
*Grade*			
1	32 (19.8)	26 (19)	6 (24)
2	96 (59.2)	83 (60.6)	13 (52)
3	32 (19.8)	26 (19)	6 (24)
NR	2		
*Surrogate subtype*			
Luminal A	70 (43.2)	63 (46)	7 (28)
Luminal B	55 (33.9)	42 (30.7)	13 (52)
ERBB2-enriched	21 (12.9)	18 (13.1)	3 (11.5)
Triple negatif	15 (9.2)	13 (9.4)	2 (8)
NR	1		
*Ki-67 index*			
< 20	107 (66)	96 (70.1)	11 (44)
≥ 20	53 (32.7)	39 (28.5)	14 (56)
NR	2	2	0
*No. of positive SLNs*			
0	137 (84.5)	137 (100)	0
1	22 (13.6)	0	22 (88)
> 2	3 (1.9)	0	3 (12)
*Pathological node status*			
pTNx	0	0	0
pTN0	137 (84.6)	137 (100)	0
pTNi+	0	0	0
pTNmi	8 (4.9)	0	8 (32)
pTN1	17 (10.5)	0	17 (68)

ER, Estrogen receptor; PgR, Progesterone receptor; SLNB, Sentinel lymph node biopsy ; pT1a, Breast tumor more than 0,1 cm but at most 0,5 cm; pT1b, Breast tumor more than 0,5 cm but at most 1 cm ; pT1c, Breast tumor more than 1 cm but atmost 2 cm, Luminal A, ER ≥ 10% and PR ≥ 10%, Luminal B, ER ≥ 10% and PR < 10%

Breast-conserving surgery was the main procedure (75.3%). Among the 52 premenopausal patients, 36 underwent breast-conserving surgery and 16 underwent mastectomy. In terms of adjuvant treatment, anti-hormonal therapy was administered in 141 of 147 hormone receptor–positive tumors (95.9%), 80.2% underwent radiotherapy, and 35.8% received adjuvant chemotherapy. Among the 130 patients treated with radiotherapy, 122 underwent whole-breast irradiation following breast-conserving surgery and 8 received post-mastectomy radiotherapy. Among node-positive patients (*n* = 25), 22 received radiotherapy, while 3 did not receive adjuvant radiotherapy after mastectomy. Regional nodal irradiation was performed in 20 patients, including 17 node-positive cases. Treatment characteristics are presented in Table 2.

**Table 2:** Final surgical treatment and recommended adjuvant therapy

Treatment	Patients, *n* = 162 (%)	N0 *n* = 137 (%)	N + *n* = 25 (%)
*Surgery*			
Breast-conserving	122(75.3)	104 (75.9)	18 (72)
Breast-conserving, SLNB	120 (74)	103 (75.1)	17 (69)
Breast-conserving, SLNB, andAD	2(1.2)	1 (0.7)	1 (4)
Mastectomy, SLNB, andAD	40 (24.7)	33 (24.1)	7 (28.0)
*Anti-hormonal*			
Yes	141 (87)	118 (86.1)	23 (92)
No	21 (13)	19 (13.9)	2 (8)
*Breast radiotherapy*			
Yes	130 (80.2)	108 (78.9)	22 (88)
No	32 (19.8)	29 (21.1)	3 (12)
*Chemotherapy*			
Yes	58 (35.8)	41 (29.9)	16 (64)
No	104 (64.2)	96 (70.1)	9 (36)
*Traztuzumab*			
Yes	20 (12.3)	17 (12.4)	3 (12)
No	142 (87.7)	120 (87.6)	22 (88)

SLNB, sentinel lymph node biopsy ; AD, axillary dissection

No differences in the application of postoperative systemic treatment were observed between the two groups except for chemotherapy among Limited-information MDT population.

### Multi-disciplinary team

Following Limited-information MDT discussion of the 25 patients, adjuvant chemotherapy was recommended for eight patients, Oncotype DX testing was requested for 11, and in six cases, neither chemotherapy nor genomic testing were considered necessary.

After additional information on axillary surgery was reviewed, in the group without further treatment or investigations, four patients underwent Oncotype DX testing, one was directly recommended for chemotherapy, and one remained in this category without additional intervention.

In the Oncotype group, five patients still required Oncotype DX testing, five were directly recommended for chemotherapy, and one did not require either chemotherapy or genomic testing.

Furthermore the eight patients initially recommended for chemotherapy remained in the same treatment category.

**Fig. 2:**
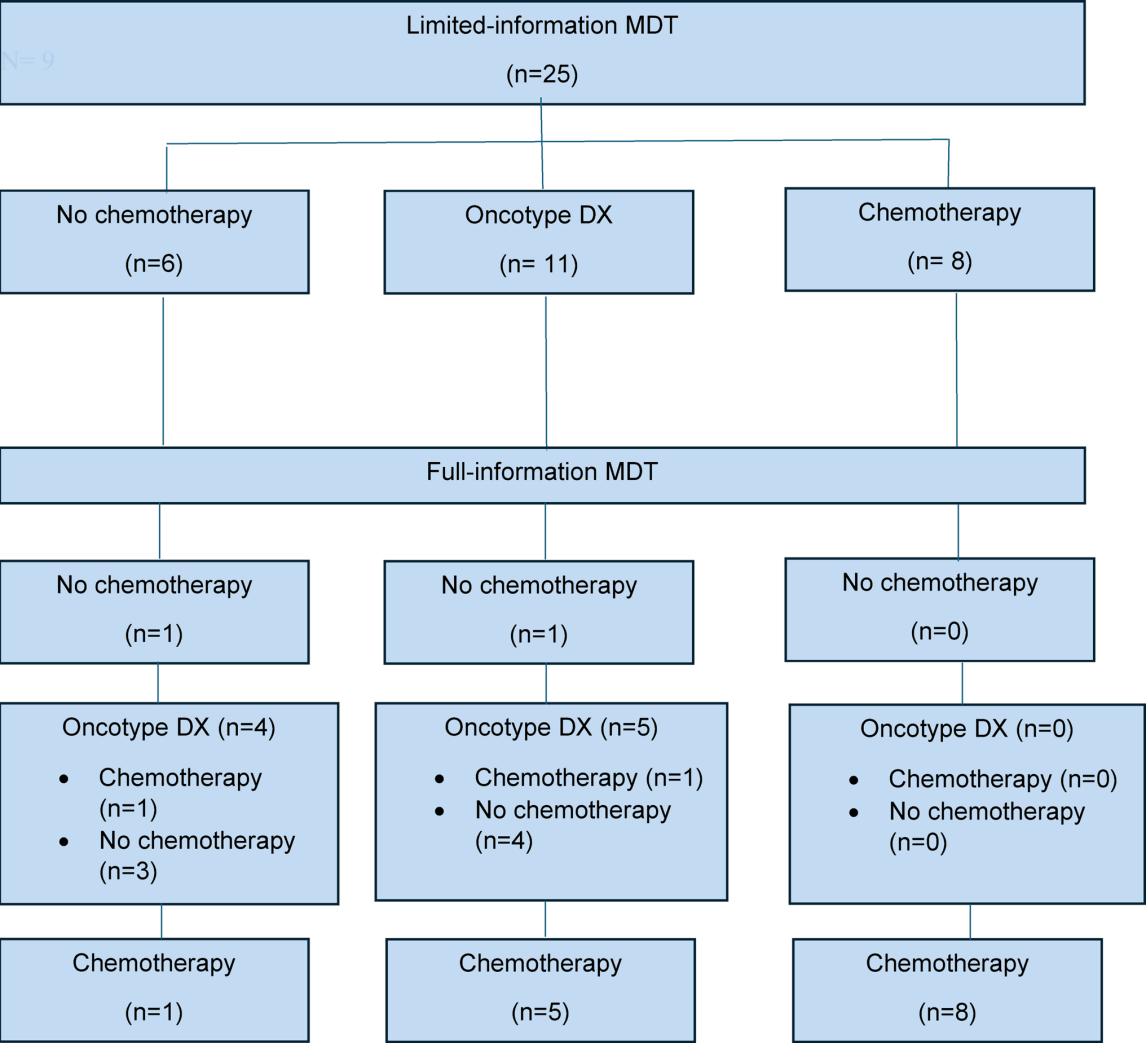
Multidisciplinary team

MDT characteristics are presented in Fig. 2.

### Final treatment

 No difference was observed between the limited-information and full-information MDT groups regarding the absence of indication for adjuvant chemotherapy. In the group no chemotherapy (*n* = 6) after the limited information MDT, two patients received chemotherapy after full information MDT (when the nodal status was known).

In the group oncotype (*n* = 11) after the limited information MDT, five patients were recommended to have a multigene expression based test (oncotype DX).

 In the Limited-information MDT the decision to administer chemotherapy was significantly lower (*p* < 0.01). In the limited-information MDT group, 8 patients were recommended to receive chemotherapy without the use of a multigene expression based test (oncotype DX). In the limited MDT, the recommended multigenerational testing for patients was not performed due to the retrospective nature of the analysis, and therefore the results were not available. In comparison, 16 patients were recommended chemotherapy in the full-information MDT group.

Contingency table is presented in Table 3.

**Table 3:** Treatment decisions according to limited vs. full MDT discussion

Limited MDT / Full MDT	No chemotherapy	Chemotherapy
Chemotherapy	0	8
Oncotype DX	5	6
No chemotherapy	4	2

MDT, Multi-disciplinary team

### Complications

Among the 162 patients included, 27 postoperative complications were recorded. All events were classified as Clavien–Dindo grade I or II. 13 patients developed seromas of the breast and/or axilla, six cases of arm or shoulder mobility restriction, five cases of axillary web syndrome (cording), two wound infections, two hematomas, one nerve injury, and three cases of arm or shoulder pain. All complications were short-term and were classified as grade I, except those requiring antibiotic therapy or simple needle aspiration, which were classified as grade II.

When stratified according to axillary surgery, 26 complications occurred after sentinel lymph node biopsy (SLNB) and 1 complication occurred after axillary lymph node dissection (ALND).

Regarding our 25 patients from our limited-information MDT, we observed three post-operative complications. All the complications were short-term complications. Two patients developed shoulder mobility limitations, which resolved after physical therapy, and one patient experienced a non-recurrent lymphocele requiring a single aspiration.

### Follow-up

The median follow-up for disease and vital status assessment was 3.6 years.

There were no deaths or recurrences occurred regarding disease-free survival in the 25 patients from our limited-information MDT patients, making a survival analysis not feasible.

## Discussion

Our findings suggest that omission of surgical axillary staging in selected patients with pT1 breast cancer does not compromise oncologic outcomes or adjuvant treatment planning. Rather, it supports treatment de-escalation and increased use of genomic assays such as Oncotype DX, promoting a more personalized and less invasive approach to early-stage breast cancer management.

Our cohort shares key inclusion characteristics with the SOUND and INSEMA trials, notably a predominance of hormone receptor–positive/HER2-negative tumors and a very limited proportion of triple-negative cancers, reflecting a low-risk population suitable for axillary de-escalation. The false-negative rate of preoperative axillary ultrasound in our study was 15% (25/162), consistent with SOUND (14.9%) and INSEMA (17%), confirming a reproducible rate across cohorts of clinically node-negative pT1 disease.

According to the SEER (Surveillance, Epidemiology, and End Results) database, a widely used reference cohort, approximately 66.6% of T1 breast cancers are hormone receptor–positive/HER2-negative, 10.8% are triple-negative, 9.7% are hormone receptor–positive/HER2-positive, and 4.3% are hormone receptor–negative/HER2-positive subtypes. Our cohort exhibits a similar distribution of molecular subtypes, confirming its representativeness of the general T1 breast cancer population across all molecular types (Howlader et al. 2018). Regarding surgical management, it is important to note that the SOUND trial and INSEMA trial included only patients undergoing breast-conserving surgery, precluding any direct comparison with our cohort, which also included patients treated with mastectomy.

The higher proportion of mastectomy observed in our study likely reflects differences in tumor characteristics, particularly the presence of extensive ductal carcinoma in situ (DCIS), which may limit the feasibility of breast-conserving surgery (Fisher et al. 2002).

This is consistent with the design of the SOUND trial, which excluded patients with extensive intraductal components, while the INSEMA trial included less than 1% of patients with associated DCIS. Therefore, our population may better reflect real-world clinical practice, where multifocality and extensive DCIS remain important factors influencing surgical decision-making and may contribute to a higher use of mastectomy.

The lower rate of adjuvant radiotherapy observed in our cohort is mainly explained by the higher proportion of patients treated with mastectomy. In our center, we adhere to European guidelines regarding indications for breast irradiation, namely, radiotherapy is systematically indicated after breast-conserving surgery, whereas it is not indicated after mastectomy in the absence of high-risk factors. Consequently, the higher number of mastectomies in our cohort directly contributed to the lower proportion of patients receiving adjuvant radiotherapy (Loibl et al. 2024).

Our findings show that the limited-information MDT led to fewer direct chemotherapy prescriptions and resulted in more oncotype tests being performed in cases of doubt compared to real-life decisions made with full axillary information.

This tendency toward treatment de-escalation contrasts with earlier evidence, such as the historical meta-analysis by the Early Breast Cancer Trialists’ Collaborative Group (EBCTCG) (Polychemotherapy for early 1998). This meta analyses demonstrated a survival benefit with adjuvant chemotherapy even in early-stage tumors, including pT1 cancers. In that analysis, chemotherapy led to a 7% absolute improvement in 10-year overall survival in node-negative patients under 50. However, those results were based on trials conducted before 1990, in a context where tumor biology was less well understood and molecular profiling tools like Oncotype DX were not available.

In light of the results of the SOUND and INSEMA trials, and supported by our smaller institutional cohort, our findings suggest that therapeutic de-escalation of axillary surgery and chemotherapy may be safely considered in carefully selected patients, particularly those with pT1 tumors and negative axillary ultrasound evaluation. While our sample size is limited, these data strengthen the growing evidence supporting a more conservative approach to axillary management in this setting.

In our cohort, the limited-information MDT more frequently used Oncotype DX to support adjuvant decisions when axillary status was unknown, which contributed to fewer chemotherapy recommendations. This approach is supported by health-economic data: a Dutch budget impact analysis estimated that incorporating Oncotype DX in hormone receptor–positive, node-negative breast cancer reduces total costs by about €13.125 per patient compared with no genomic testing (Jongh et al. 2022). Other validated assays—such as MammaPrint, Prosigna, and EndoPredict—show comparable prognostic performance in stratifying recurrence risk. Therefore, the choice of genomic test largely reflects institutional preference, availability, and cost considerations, rather than significant differences in clinical utility.

In our study, Oncotype DX–guided chemotherapy decisions were adapted to menopausal status, using a recurrence score cut-off of 15 for premenopausal women and 25 for postmenopausal women. This strategy is supported by TAILORx, which showed no chemotherapy benefit for women over 50 years with scores < 25, while younger women (≤ 50 years) with scores 16–25 may still benefit (Sparano et al. 2018) Similarly, RxPONDER demonstrated no benefit in postmenopausal patients with 1–3 positive nodes and scores ≤ 25, whereas premenopausal women derived benefit from chemotherapy (Han et al. 2025). This approach enables personalized treatment decisions that balance efficacy and toxicity according to hormonal and biological context. In addition, our results suggest that the availability of axillary staging information may influence the use of genomic testing in clinical practice. Chemotherapy was more frequently prescribed without prior Oncotype DX testing in the full MDT group compared to the limited MDT group, whereas the use of Oncotype DX was higher when nodal status was unknown. Notably, a substantial proportion of patients receiving chemotherapy without genomic testing were premenopausal, suggesting that knowledge of nodal status may prompt clinicians to favor upfront chemotherapy in this subgroup without additional molecular stratification. Menopausal status therefore appears to modulate the clinical impact of axillary staging on systemic treatment decisions.

No cancer-related recurrences or deaths were observed during follow-up, consistent with the favorable prognosis of pT1 tumors and supported by large retrospective data (Houvenaeghel et al. 2014) including 8.100 operated women, among whom 5.423 had pT1 tumors, that reported 5-year recurrence-free survival rates by subtype for pT1 tumors: 94.7% for luminal A, 82.3% for luminal B HER2−, 91.4% for luminal B HER2+, 89.0% for triple-negative, and 90.2% for HER2 + non-luminal tumors. However, given the limited sample size and short follow-up, particularly in luminal tumors with known late recurrence risk, these results should be interpreted cautiously.

A large EBCTCG meta-analysis showed that luminal A breast cancers carry a substantial risk of late recurrence, with many events occurring 5–20 years after diagnosis, whereas triple-negative and HER2-positive subtypes tend to recur earlier, mainly within the first 3 years (Pan et al. 2017). Consequently, the absence of oncologic events in our cohort should be interpreted with caution, and larger, long-term prospective studies are needed to confirm the safety of the evaluated therapeutic strategies.

Omitting SLNB may significantly reduce postoperative morbidity without compromising oncological outcomes in selected patients (Aaronson et al. 1993). The randomized INSEMA trial demonstrated a clinically and statistically significant reduction in arm-related symptoms—including pain, swelling, and reduced mobility—up to 18 months in patients who did not undergo SLNB, indicating a sustained functional burden attributable to SLNB rather than breast surgery itself (Reimer et al. 2025). Similar results have been reported in large prospective and real-world cohorts. The ACOSOG Z0010 study showed that, despite being less invasive than axillary lymph node dissection, SLNB remains associated with both short- and long-term complications, including seroma, paresthesia, reduced range of motion, and lymphedema, with seroma being the most frequent event (Gunn et al. 2016). Persistent shoulder mobility impairment up to two years after surgery has also been reported in a systematic review by Torres Lacomba et al. (Verbelen et al. 2014).

Although generally less severe than complications following axillary dissection, these sequelae remain clinically relevant, particularly in elderly or frail patients and in younger, physically active women, supporting an individualized indication for SLNB in patients with a low expected nodal risk (Wilke et al. 2006). Contemporary evidence further supports axillary de-escalation: both the BOOG 2013-08 randomized trial and real-world INSEMA data confirm the feasibility of omitting SLNB in carefully selected patients with favorable tumor biology treated with breast-conserving surgery and radiotherapy. However, real-world INSEMA analyses indicate that nodal status may still influence adjuvant treatment decisions beyond strictly low-risk subgroups, underscoring the need for biology-driven, patient-tailored decision-making supported by complementary tools (Tauber et al. 2025, Wintraecken et al. 2025).

However, these findings should be interpreted in the context of several limitations, including the relatively small sample size, the heterogeneity of the population (age, surgical procedures, and biological subtypes), and the limited follow-up duration, which may impact the robustness and generalizability of subgroup analyses.

These findings support the ongoing paradigm shift from anatomical to biological treatment decision-making in early breast cancer.

## Conclusion

Our findings suggest that omission of surgical axillary staging in carefully selected patients with clinically node-negative (cN0), small (pT1) breast cancer does not compromise adjuvant treatment decisions or short-term oncologic outcomes. This approach appears to be supported by a combination of factors, including negative preoperative axillary imaging, small tumor size, favorable tumor biology, particularly hormone receptor–positive/HER2-negative subtypes and menopausal status. In line with current evidence, omission of axillary staging is more clearly supported in postmenopausal patients, in whom systemic treatment decisions are less dependent on nodal status. In contrast, in premenopausal patients and those with more aggressive tumor biology, axillary staging remains more relevant, as nodal status may still influence adjuvant treatment decisions. In these cases, genomic assays such as Oncotype DX may help guide systemic therapy independently of axillary surgery. Overall, these findings support a personalized, biology-driven approach to axillary management in early-stage breast cancer, consistent with the ongoing shift toward treatment de-escalation while maintaining oncologic safety.

## Data Availability

The datasets generated and/or analysed during the current study are not publicly available due to patient confidentiality but are available from the corresponding author on reasonable request.
